# Optimizing CaO-based CO_2_ adsorption: Impact of operating parameters

**DOI:** 10.1016/j.isci.2025.113127

**Published:** 2025-07-16

**Authors:** Chengzhuang Zhang, Jia Fang, Zhiqiang Han, Peng Chen, Xilong Xu, Kejian Wang, Yunxi Shi, Jianxiong Liao

**Affiliations:** 1 Key Laboratory of Fluid and Power Machinery, Ministry of Education, School of Energy and Power Engineering, Xihua University, Chengdu, China; 2 Key Laboratory of Fluid Machinery and Engineering, School of Energy and Power Engineering, Sichuan Province, Xihua University, Chengdu, China; 3 Engineering Research Center of Intelligent Space Ground Integration Vehicle and Control, Ministry of Education, Xihua University, Chengdu, China; 4 School of Automobile and Transportation, Xihua University, Chengdu, China; 5 School of Automotive and Traffic Engineering, Jiangsu University, Zhenjiang, China

**Keywords:** applied sciences, chemistry, environmental science

## Abstract

CaO-based adsorbents present a promising approach for industrial CO_2_ emissions reduction. This study systematically investigates the influence of adsorption temperature, adsorbent mass, CO_2_ concentration, and flow rate on CO_2_ capture efficiency using commercial CaO via thermogravimetric analysis (TGA). Optimal adsorption performance, achieving a capacity of 0.62 g/g and a rate exceeding 0.14 g/g/min, was observed at 750°C, 7 mg mass, 20% CO_2_, and 20 mL/min flow. Characterization (SEM, BET, and FTIR) indicated that 750°C preserved a hierarchical pore structure (24.64 m^2^/g surface area, 0.1026 cm^3^/g pore volume, and 7.08 nm average pore size), mitigating sintering and pore blockage while confining carbonation to surface layers. Kinetic modeling confirmed chemisorption dominance (pseudo-second-order). These findings elucidate fundamental mass/heat transfer limitations, supporting the development of enhanced CaO-based carbon capture, utilization, and storage (CCUS) technologies.

## Highlights

• Optimal CO_2_ capture: 0.62 g/g at 750°F, 20% CO_2_, 20 mL/min, and 7 mg CaO
• Hierarchical pore structure preserved (24.64 m^2^/g SSA, 0.1026 cm^3^/g) under optimal conditions
• Adsorption kinetics conform to pseudo-second-order model (R^2^ > 0.99), chemisorption dominant
• Establishes mechanistic basis for scaling low-cost CaO-based CCUS technologies

## Graphical abstract

**Figure:**
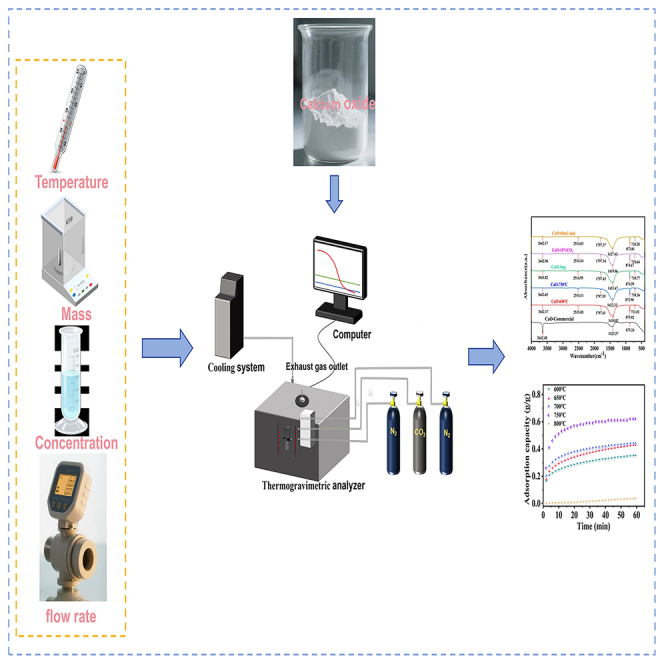

## Introduction

Since the early 21st century, substantial greenhouse gas emissions have driven the ongoing degradation of Earth’s climate and environment.^1^ As global warming resulting from the greenhouse effect continues to seriously affect human life, carbon capture, utilization, and storage (CCUS) has become increasingly important as one of the most promising strategies for reducing industrial CO_2_ emissions.^2^ At the initial stage of CCUS, carbon capture serves as a pivotal technology that significantly influences the efficiency and cost of subsequent CO_2_ utilization and storage. The three primary carbon capture techniques are solid adsorption, liquid absorption, and membrane separation.^3^ Liquid-phase absorption processes, particularly those utilizing amine-based solvents, such as monoethanolamine (MEA), demand significant thermal energy for the regeneration of conventional amine solutions, with energy consumption values reaching up to 2.5–4.0 GJ/t of CO_2_.^4^^,^^5^^,^^6^^,^^7^ Membrane separation technology is limited by the selectivity and permeability of membranes and requires high-pressure operation (5–10 bar). Additionally, membrane fouling significantly reduces the service life of industrial membrane modules.^8^^,^^9^^,^^10^^,^^11^ Solid adsorbents, which are essential to adsorption processes, primarily include zeolites, activated carbon (AC), metal-organic frameworks (MOFs), and alkali metals.^12^ Although activated carbon can effectively adsorb organic molecules and CO_2_,^13^^,^^14^^,^^15^ it has low CO_2_/N_2_ selectivity and its specific surface area declines by 30–50% after multiple adsorption-desorption cycles, making it difficult to treat flue gas with low CO_2_ concentration and high humidity.^16^ MOFs suffer from several drawbacks, including a significant reduction in adsorption capacity under high humidity, high synthesis costs (up to $800/kg for ZIF-8), and insufficient mechanical stability (with >30% fragmentation during cyclic testing).^17^^,^^18^ At low pressure (1 bar), the CO_2_ adsorption capacity of zeolite is only 0.2–0.4 g/g. Under high humidity (RH > 50%), this capacity further decreases by 50–70%. Moreover, the energy consumption for regeneration via temperature-swing operation can be as high as 1.2–1.8 GJ/t CO_2_.^19^^,^^20^ Alkali-metal adsorbents, such as potassium carbonate (K_2_CO_3_), often exhibit sluggish reaction kinetics, deliquescence-induced agglomeration, and rapid loss of adsorption capacity.^21^^,^^22^ In comparison to alternative adsorbents, the CaO adsorbent exhibits a number of advantageous characteristics. (1) High capacity and heat-integrated carbonation: The carbonation reaction (CaO + CO_2_ → CaCO_3_) achieves a theoretical capacity of 786 mg/g at 600°C–900°C. By utilizing industrial waste heat, regeneration energy consumption is reduced to 0.8–1.8 GJ/t of CO_2_, representing a 30–50% reduction compared to amine-based processes.^23^ (2) Humidity tolerance and selectivity: Unlike many solid sorbents, CaO actually benefits from modest water vapor. Introducing a moderate humidity (e.g., RH ≈ 10%) promotes surface hydration and significantly accelerates the carbonation kinetics. In practice, CaO retains over 85% of its CO_2_ capacity even at RH >50%.^24^^,^^25^ (3) Cost-effectiveness: The raw material (limestone/CaCO_3_) is extremely abundant and inexpensive (on the order of US$10–20 per ton), enabling large-scale production of CaO-based sorbents. More importantly, CaO sorbents can be cycled hundreds of times with modest loss in performance.^26^^,^^27^ (4) Kinetic advantage: low activation energy (50–80 kJ/mol) ensures rapid CO_2_ uptake, achieving adsorption rates 5–10 times faster than K_2_CO_3_ in humid flue gases.^28^^,^^29^ These advantages position CaO-based adsorbents as ideal candidates for industrial carbon capture, utilization, and storage (CCUS) systems.

Extensive experimental studies have investigated the CO_2_ adsorption properties of CaO-based adsorbents, focusing on high-temperature thermal activation,^30^^,^^31^ hydration treatments,^32^^,^^33^ synthetic porous CaO fabrication,^34^^,^^35^ and doping with metal oxides.^36^^,^^37^^,^^38^^,^^39^^,^^40^^,^^41^ However, systematic analyses of operational parameter impacts (e.g., temperature, gas flow rate, and CO_2_ concentration) on adsorption efficiency remain limited. Some scholars have investigated the effects of operating parameters within neural network frameworks.^42^^,^^43^^,^^44^ However, their work remains limited. For example, Wang et al. ^45^ employed machine learning (XGBoost) to predict CaO capture performance under varying inlet temperatures, flow rates, CO_2_ mass fractions, and adsorbent bed heights, these studies lacked mechanistic explanations for parameter-driven performance changes. This study addresses this gap by integrating laboratory-scale experiments to develop a reaction kinetic model for CaO-based CO_2_ capture using experimental data, thereby gaining insight into the adsorbent’s capture efficiency under varying conditions and elucidating the mass and heat transfer mechanisms in the CO_2_ capture process. These findings provide a foundation for optimizing design parameters and enhancing process efficiency.

In this study, the CO_2_ capture process by CaO was first simulated using a thermogravimetric test (TGA) rig to evaluate the effects of four key parameters—adsorption temperature, adsorbent mass, CO_2_ concentration, and CO_2_ flow rate—on CO_2_ capture efficiency. Subsequent characterization via scanning electron microscopy (SEM), Brunauer-Emmett-Teller (BET) surface area analysis, and fourier transform infrared spectroscopy (FTIR) is performed to investigate how different operating conditions influence capture performance, clarify the adsorption mechanism, and identify the underlying causes of parameter effects. Optimal CaO adsorption performance is achieved under the following conditions: 750°C, 7 mg adsorbent mass, 20% CO_2_ concentration, and 20 mL/min CO_2_ flow rate. By optimizing the operating conditions of the capture process, this work enhances both the efficiency and economic viability of CO_2_ capture, providing a foundation for further research on more effective CaO-based adsorbents for CO_2_ capture and utilization.

### Experimental and computational

**Figure 1:**
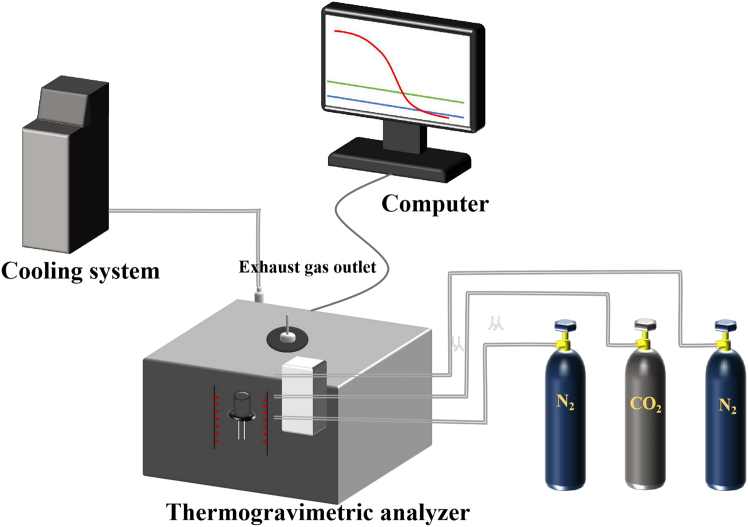
Schematic diagram of the test rig

In this study, a thermogravimetric analyzer (Model TG209F3) is employed to monitor and record in real time the mass change of the sample as a function of time/temperature under different gas atmospheres. The thermogravimetric analyzer is considered an effective technique for analyzing the thermal cracking behavior of samples due to its good dynamic response.^46^^,^^47^ The schematic diagram of the test rig is shown in Figure 1.

The thermogravimetric test bench is equipped with computer software for thermogravimetric analysis, a high-precision balance, a constant temperature water bath (to ensure the stability of the signal of the balance), a flowmeter, heating elements and gas cylinders (to provide the gas atmosphere), and the main technical parameters. Table 1 illustrates the specifications of the thermogravimetric analyzer. The thermogravimetric test bench employs a specific gas atmosphere, whereby the high-precision balance records the change in mass of the sample in real time at a given temperature and under a specified gas atmosphere.^48^^,^^49^

**Table 1:** Technical parameters of the thermogravimetric analyzer

Items	Parameters
Balance Measurement Response Range (g)	0∼2
Balance sensitivity (μg)	0.1
Temperature rise rate (°C/min)	0.001–100
Crucible size (mm)	Φ6.8 × 7.4
Crucible volume (μL)	268
Temperature range (°C)	indoor temperature ∼1000

SEM analysis: surface morphology and microstructure are characterized using a Thermo Scientific Aprea 2C scanning electron microscope. Prior to imaging, samples are sputter-coated with a 5 nm gold-palladium layer to enhance conductivity. BET analysis: prior to BET analysis, degassing is performed at 300°C for 4 h. Specific surface area, pore volume, and pore size distribution are determined via nitrogen adsorption-desorption isotherms at 77 K using a Micromeritics ASAP 2460 analyzer. Surface area is calculated using the Braeuer-Emmett-Teller (BET) method, while pore size distribution is derived from the Barrett-Joyner-Halenda (BJH) model applied to the adsorption branch. FTIR spectra are recorded on a Thermo Scientific Nicolet iS5 spectrometer (Thermo Fisher Scientific, USA) in the mid-infrared range (4000–400 cm^−1^). Samples are analyzed using the KBr pellet method, wherein powdered specimens are homogenized with spectroscopic-grade potassium bromide and pressed into transparent disks. Spectra are collected at 4 cm^−1^ resolution with 32 cumulative scans.

In order to measure the effect of different parameters on the performance of carbon dioxide adsorption by CaO more accurately, commercial CaO (the material is purchased from Aladdin Biochemical Company, Shanghai, China) is used in this experiment, and its characteristic parameters are shown in Table 2.

**Table 2:** Physical properties of adsorbent

Adsorbent	Molecular mass	Metal’s basis
CaO	56.08	99.95%

The gas mixture simulates pre-purified flue gas (post-desulfurization and final drying), as untreated flue gas cannot be effectively separated using a single-layer adsorbent. Consequently, the experimental gas environment is supplied via pre-mixed gas cylinders to replicate industrial carbon capture conditions.^50^ The TGA is first heated from room temperature to 45°C, held for 10 min, and then ramped at 40°C/min under N_2_ to a predefined temperature. Upon reaching the target temperature, the gas is switched to 20% CO_2_/N_2_, and the temperature is maintained for 1 h to conduct adsorption measurements. Post-adsorption, the system is stabilized under N_2_ to terminate the experiment. To assess stability, 20 adsorption-desorption cycles are conducted under optimal parameters: 7 mg of CaO is subjected to 1-h adsorption in a 20% CO_2_ stream at 20 mL/min, followed by 30-min desorption under pure N_2_. The temperature is maintained at 750°C throughout the entire process.

The experiment is designed with four test groups to investigate the effects of different operating parameters on CaO-mediated CO_2_ adsorption, employing the controlled-variable method: (1) Taslima et al.^51^ investigated CaO-based adsorbents via TGA across temperatures of 450°C–800°C, while Liu et al.^52^ reported optimal adsorption performance for a calcium carbide-based adsorbent at 650°C, with both studies desorbing at 800°C. To further explore temperature effects, five test points (600, 650, 700, 750, and 800°C) are selected. Experiments are conducted under a 20% CO_2_/80% N_2_ gas atmosphere, with a CO_2_ flow rate of 20 mL/min, 7 mg CaO mass, and a heating rate of 40°C/min (2) Using a TGA system, the adsorbent is tested as a powder in a Φ6.8 × 7.4 mm crucible, which typically accommodates a sample mass of 20–40 mg. For commercial CaO, 20 mg fills approximately half of the crucible, whereas Karl O. Albrecht et al.^53^ used a maximum mass of 11 mg in their thermogravimetric study of CaO-based CO_2_ adsorbents. To accurately assess the effect of adsorbent mass on CaO’s adsorption properties, five mass points—3, 5, 7, 9, and 11 mg—are tested under a 20% CO_2_/80% N_2_ gas atmosphere. Experimental conditions included a CO_2_ flow rate of 20 mL/min, an adsorption temperature of 750°C, and a heating rate of 40°C/min (3) Taslima et al.^51^ investigated the effects of operating parameters on CaO-based CO_2_ adsorption at 10% and 15% CO_2_/N_2_ concentrations, while Liu et al.^52^ used a 20% CO_2_/80% N_2_ mixture and Ahmed et al. employed a 10% CO_2_/N_2_ atmosphere. To investigate the effect of CO_2_ concentration, three gas mixtures—10%, 15%, and 20% CO_2_/N_2_—are tested in the experiments. Experiments are conducted under a constant CO_2_ flow rate of 20 mL/min, adsorption temperature of 750°C, CaO mass of 7 mg, and heating rate of 40°C/min (4) Nattha et al.^54^ demonstrated through fluidized bed experiments that laminar flow (Re < 20) is maintained at 20–40 mL/min, mitigating uneven mass transfer caused by turbulence, when investigating the effect of flow rate on the fluidization state of adsorbent particles. Therefore, the effects of three CO_2_ flow rates—20, 30, and 40 mL/min—are tested under a 20% CO_2_/80% N_2_ gas atmosphere, with the adsorption temperature, CaO mass, and heating rate set at 750°C, 7 mg, and 40°C/min, respectively. To minimize experimental error, each parameter set is replicated twice, and mean values are calculated for analysis.The experimental data is listed in Table 3.

**Table 3:** Summary of characteristic parameters of all experiments in this study

	Adsorption temperature (°C)	Sample mass (mg)	Carbon dioxide concentration (%)	Carbon dioxide flow rate (mL/min)	Adsorption time (min)	Percentage of onset of adsorption (%)	Percentage of end of adsorption (%)	Carbon dioxide adsorption capacity (g/g)
1–2	600	7	20	20	60	85.76	115.77	0.35
3–4	650	7	20	20	60	85.89	123.07	0.43
5–6	700	7	20	20	60	86.78	124.88	0.44
7–8	750	7	20	20	60	78.10	126.32	0.62
9–10	800	7	20	20	60	76.03	78.79	0.04
11–12	750	3	20	20	60	108.32	157.32	0.45
13–14	750	5	20	20	60	91.28	135.51	0.48
15–16	750	7	20	20	60	78.10	126.32	0.62
17–18	750	9	20	20	60	92.06	136.80	0.49
19–20	750	11	20	20	60	87.91	135.98	0.51
21–22	750	7	10	20	60	78.68	120.88	0.54
23–24	750	7	15	20	60	78.05	125.06	0.60
25–26	750	7	20	20	60	78.10	126.32	0.62
27–28	750	7	20	20	60	78.10	126.32	0.62
29–30	750	7	20	30	60	78.74	125.64	0.60
31–32	750	7	20	40	60	79.54	125.09	0.57

**Figure 2:**
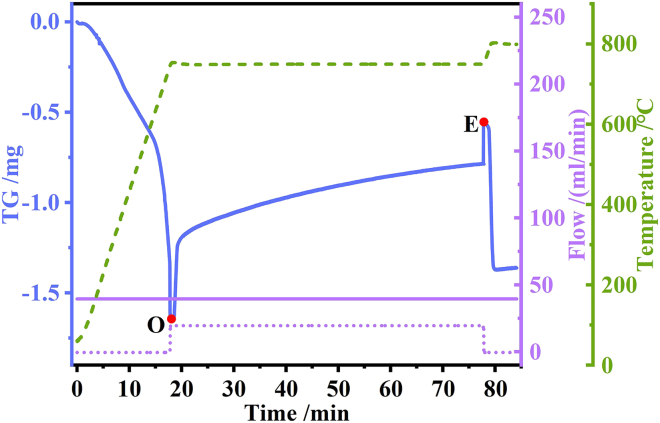
Adsorption desorption curves of carbon dioxide adsorption by CaO

The adsorbed mass per unit mass of adsorbent on the adsorbent, designated as adsorption capacity C (unit: g/g), is calculated using the adsorbent cyclic adsorption/desorption curves. The adsorption rate S (unit: g/g/min) is obtained by deriving the derivation of the adsorption capacity, that is, the amount of change in adsorption capacity C per unit time. Figure 2 illustrates the adsorption and desorption curve of the CaO adsorbent at point O. At this juncture, nitrogen is substituted for the 20% CO_2_ mixture, thereby initiating the adsorption of CO_2_; conversely, at point B, the 20% CO_2_ mixture is replaced by nitrogen, which represents the conclusion of the adsorption process. Between point O and point E, the adsorption of CO_2_ by the adsorbent is evident; subsequently, after point E, the desorption of CO_2_ becomes apparent. The CO_2_ adsorption capacity of the adsorbent is calculated according to the following formula:

(Equation 1) $$C=\frac{M_{E}-M_{O}}{M_{O}}*100\%$$

(Equation 2) $$S=\frac{dc}{dx}*100\%$$

M_O_-- Percentage mass of sample that adsorbs starting point O,%;

M_E_-- Percentage mass of sample at adsorption endpoint E,%;

The CO_2_ adsorption kinetics is analyzed using pseudo-first-order and pseudo-second-order models. The pseudo-first-order model (Equation 3) assesses physical adsorption dominance by describing the proportionality between adsorption rate and available sites^55^:

(Equation 3) $$\ln\left(q_{e}-q_{t}\right)=\ln\;q_{e}-k_{1}$$

Where q_t_ and q_e_ are adsorption capacities (mmol·g^−1^) at time t (min) and equilibrium, respectively, and k1 is the rate constant (min^−1^).

The pseudo-second-order model (Equation 4) describes chemisorption-controlled processes, where the adsorption rate relates quadratically to site availability^55^:

(Equation 4) $$\frac{t}{q_{t}}=\frac{k_{2}}{{q_{e}}^{2}}+\frac{t}{q_{e}}$$

where k_2_ is the rate constant (g·mmol^−1^·min^−1^).

## Results and discussions

### Physical and chemical properties analysis

**Figure 3:**
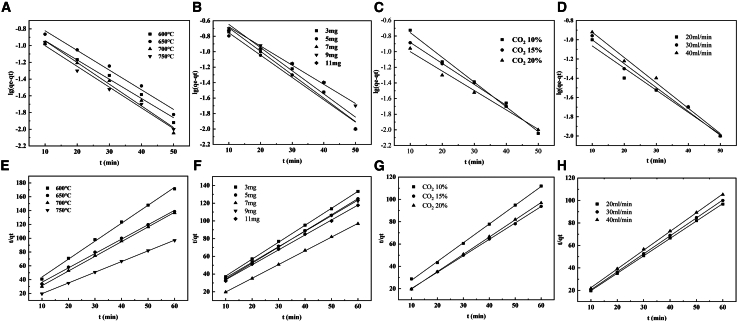
First-order kinetic fitting curves under different operating parameters (A) temperature；(B) mass, (C) CO_2_ concentration, (D) CO_2_ flow rate; second-order kinetic fitting curves: (E) temperature, (F) mass, (G) CO_2_ concentration, (H) CO_2_ flow rate.

The CO_2_ adsorption kinetics of CaO-based adsorbents are evaluated using pseudo-first-order and pseudo-second-order models (Figure 3). Both models yielded high correlation coefficients (Table 4), with the pseudo-second-order model exhibiting superior fit (R^2^ > 0.99). Notably, the equilibrium capacity (q_e_) derived from the pseudo-first-order model showed significant deviation from experimental values, while the pseudo-second-order q_e_ aligned closely with measured capacities. These results demonstrate that CO_2_ adsorption on CaO is predominantly chemisorption-controlled across all tested operating conditions.^56^

**Table 4:** Fitting parameters under different kinetic models under different adsorption conditions

Samples	Pseudo-first order kinetic model			Pseudo-second order kinetic model		
	q_e_ (g·g^−1^)	k_1_ (s^−1^)	R^2^	q_e_ (g·g^−1^)	K_2_ (g·g^−1^·s^−1^)	R^2^
600°C	5.1436	0.0530	0.9775	0.3648	0.3791	0.9975
650°C	3.8640	0.0542	0.9765	0.4404	0.2803	0.9974
700°C	4.8254	0.0597	0.9784	0.4504	0.4492	0.9993
750°C	5.6504	0.0571	0.9843	0.6354	0.5626	0.9999
3 mg	2.4426	0.0698	0.9662	0.4530	0.1963	0.9997
5 mg	2.9343	0.0664	0.9681	0.4894	0.2083	0.9996
7 mg	5.6504	0.0690	0.9843	0.6354	0.5626	0.9999
9 mg	2.6989	0.0570	0.9951	0.4998	0.1694	0.9991
11 mg	2.2258	0.0690	0.9168	0.5232	0.1697	0.9987
10%	2.7507	0.0729	0.9905	0.5553	0.2625	0.9992
15%	4.1117	0.0618	0.9975	0.6105	0.3987	0.9996
20%	5.6504	0.0571	0.9843	0.6354	0.5626	0.9999
20 mL/min	5.6504	0.0690	0.9843	0.6354	0.5626	0.9999
30 mL/min	6.8179	0.0571	0.9843	0.6156	0.5586	0.9998
40 mL/min	4.5422	0.0607	0.9911	0.5794	0.4844	0.9999

**Figure 4:**
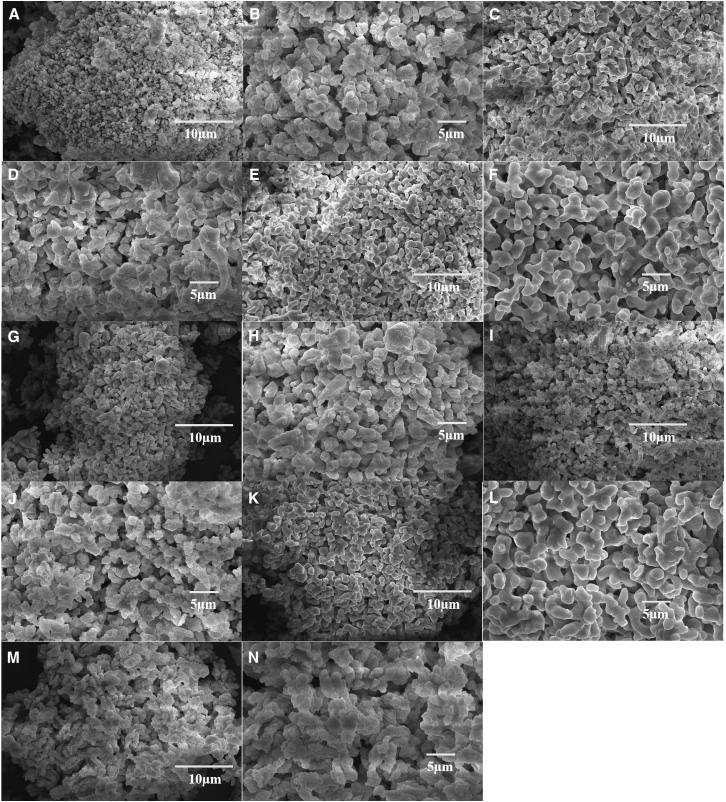
SEM images of CaO samples after CO_2_ adsorption under different operational parameters Note: (A and B): commercial CaO (10 μm, 5 μm); (C and D): 600°C-calcined CaO (10 μm, 5 μm); (E and F): 750°C-calcined CaO (10 μm, 5 μm); (G and H): 3 mg-loaded CaO (10 μm, 5 μm); (I and J): 10% CO_2_-treated CaO (10 μm, 5 μm); (K and L): CaO under 40 mL/min flow (10 μm, 5 μm); (M and N): 20-cycle CaO (10 μm, 5 μm).

Scanning electron microscopy (SEM) analysis (Figure 4) reveals that commercial CaO forms compact plate-like aggregates with a rich pore structure. Under varying adsorption conditions, the pore structures of CaO samples exhibit distinct degrees of sintering. Notably, 750°C-calcined CaO retains a three-dimensional honeycomb architecture with uniform pores, a feature directly correlated with its maximum pore volume (0.1026 cm^3^/g) and optimized mesopore diameter (7.08 nm) as confirmed by BET analysis. This contrasts sharply with other experimental conditions:

600°C-calcined CaO shows partially collapsed pore channels and fused particles, accompanied by a 9.4% reduction in pore volume (0.0938 cm^3^/g); 3 mg-loaded CaO exhibits a fractured porous network with thinned walls, leading to a 7.8% pore volume loss (0.0946 cm^3^/g); 10% CO_2_-exposed CaO undergoes extensive surface fusion and pore blockage, resulting in a 23% pore volume reduction (0.0773 cm^3^/g); CaO under a 40 mL/min flow rate suffers surface erosion and microcracking, corresponding to a 15.7% pore volume decrease (0.0865 cm^3^/g); 20-cycle treated CaO experiences severe sintering, with complete morphological restructuring into agglomerates, a 40.8% pore volume loss, and a 238% increase in average pore diameter (to 16.59 nm). The 750°C-calcined CaO maintains structural integrity, demonstrating synergistic resistance to thermal sintering, carbonate-induced expansion, and mechanical stress. This is further validated by its weak carbonate characteristic peak (1411.67 a.u.) and stable lattice vibration intensity (874.59 a.u.) at 874 cm^−1^.

To systematically investigate the impact of varying operational parameters on CO_2_ adsorption, BET characterization is performed post-adsorption. The test results are tabulated in Table 5. As evidenced by the structural characterization in Table 5, the CaO calcined at 750°C exhibits the most optimized porous architecture. It achieves a specific surface area of 24.64 m^2^/g, merely 2.3% lower than that of pristine commercial CaO (25.23 m^2^/g), while its pore volume (0.1026 cm^3^/g) represents the highest value among all samples (exceeding the 600°C sample by 9.4%, the CO_2_-10% group by 32.7%, and the cycled sample by 65.5%). Critically, its average pore size (7.08 nm) is maintained within the ideal mesoporous range, demonstrating a 36.5% reduction compared to the CO_2_-10% group (11.14 nm) and a 57.3% reduction versus the cycled sample (16.59 nm), thereby mitigating pore coarsening while avoiding diffusion limitations inherent in smaller pores (e.g., 4.90 nm in commercial CaO). The 750°C temperature effectively balances the decomposition efficiency of CaCO_3_ and the inhibition of CaO sintering, providing critical parameter references for the design of high-performance CO_2_ adsorbents.

**Table 5:** Specific surface area, average pore size, and pore volume under different operating parameters

Operating parameters	Specific surface area (m^2^/g)	Average pore size (nm)	Pore volume (cm^3^/g)
CaO-commercial	25.2256	4.9024	0.1047
CaO-600°C	24.1067	7.2405	0.0938
CaO-750°C	24.6358	7.0787	0.1026
CaO-3mg	23.9186	7.2228	0.0946
CaO-CO_2_ 10%	20.4510	11.1372	0.0773
CaO-40mL/min	23.8539	7.3019	0.0865
Cycle (20 times)	14.8596	16.5890	0.0620

**Figure 5:**
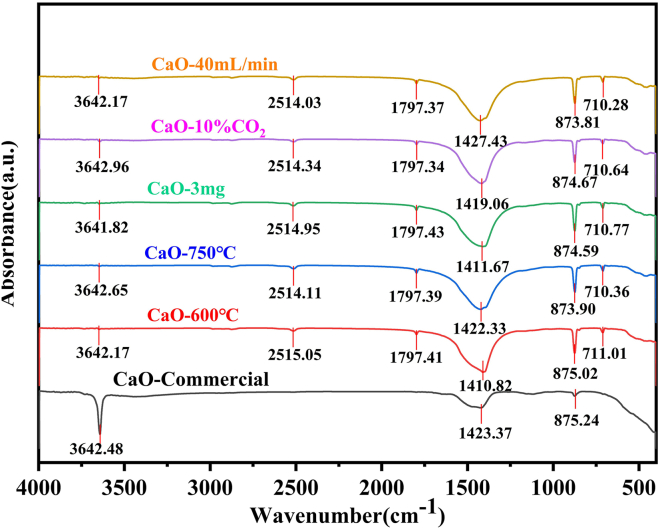
Fourier transform infrared (FTIR) spectra of CaO-based adsorbents under different operating conditions

The FTIR analysis results post-adsorption are presented in Figure 5. Under the 750°C calcination condition, the sample exhibits the least pronounced carbonization characteristics, with a peak intensity of 1411.67 a.u. at 1420 cm^−1^. This value represents a 0.82% reduction compared to the commercial CaO reference sample, which shows an intensity of 1423.37 a.u. after adsorption. This finding aligns with the optimized pore structure of the sample. Such structural features restrict carbonate formation to the near-surface region, as further evidenced by three key observations. First, surface functional groups (-OH) remain invariant across all parameter sets, with an intensity of 3642.65 ± 0.17 a.u. Second, the intensity of the 874 cm^−1^ lattice vibration increases by 0.08% at 750°C relative to 600°C, indicating enhanced structural ordering. Third, a strong correlation exists between carbonate peak intensity and pore volume loss, with a correlation coefficient R^2^ of approximately 0.93. This confirms that the 750°C parameters maximally preserve structural integrity during the adsorption process.

CO_2_ adsorption proceeds via two distinct pathways. The first pathway involves surface -OH groups reacting with CO_2_ to form bicarbonate intermediates, a process evidenced by the unchanged 3642 cm^−1^ peak. The second pathway involves lattice O^2−^ ions directly generating carbonate ions, with the intensity of the 874 cm^−1^ peak reflecting the reactivity of this pathway. The 750°C operational parameters optimize the pore structure, which has a diameter of 7.08 nm and a volume of 0.1026 cm^3^/g. This optimized structure confines the reaction to a single surface layer, thereby minimizing bulk carbonation and resulting in the lowest peak intensity of 1411.67 a.u. at 1420 cm^−1^. As a result, the contribution of the -OH pathway increases to over 60%, suppressing lattice expansion-induced passivation associated with the O^2−^ pathway.

Regarding the peaks at 2515 cm^−1^ and 1797 cm^−1^, they are assigned to two distinct phenomena. The first is a composite band of carbonate ions (CO_3_^2−^) from the combined ν_1_ and ν_3_ vibrational modes, which confirms overall carbonization. The second is a Fermi resonance band from physically adsorbed CO_2_, reflecting the pore filling capacity. Notably, the intensities of these peaks remain constant across all operating parameters. Specifically, the 2515 cm^−1^ peak ranges from 2514.03 to 2515.05 a.u. with a variation of less than 0.4%, and the 1797 cm^−1^ peak ranges from 1797.34 to 1797.43 a.u. with a variation of less than 0.005%. This consistency indicates that these vibrations are decoupled from the chemical adsorption pathway.

### Effect of temperature on the adsorption of carbon dioxide by CaO

**Figure 6:**
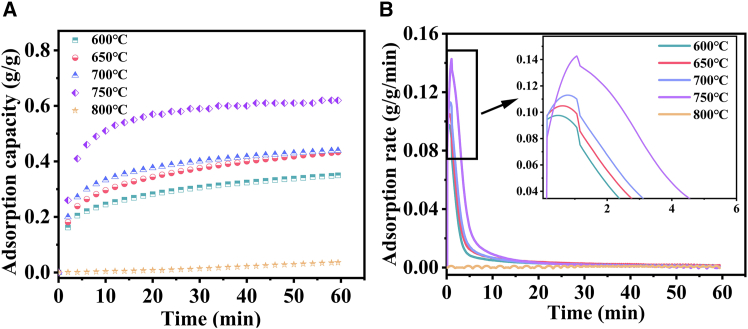
Adsorption performance of CaO at 600°C–800°C (A) adsorption capacity; (B) adsorption rate.

Figures 6A and 6B illustrate a significant positive correlation between temperature and CaO adsorption performance. As depicted in Figure 6A, the adsorption capacity exhibits a pronounced increasing trend with temperature rising from 600°C to 750°C. Correspondingly, Figure 6B demonstrates a notable acceleration in the adsorption rate with increasing temperature. At 750°C, the adsorption capacity (C) reaches 0.62 g/g, and the adsorption rate (S) exceeds 0.14 g/g/min—values significantly higher than those at other temperatures. By contrast, at 800°C, both the adsorption capacity and rate decline to their minimum values, approaching zero, which aligns with the typical desorption temperature of CaO (≈800°C).^57^ The optimal adsorption performance at 750°C can be attributed to two key factors: lower temperatures slow the reaction rate, limiting adsorption capacity, whereas higher temperatures promote CaO particle sintering, reducing available surface area; the CaO pore architecture at 750°C is optimize.^58^^,^^59^^,^^60^ In summary, increasing temperature enhances CaO’s CO_2_ adsorption capacity and rate, with 750°C identified as the optimal condition for subsequent experimental design.

### Effect of adsorbent quality on carbon dioxide adsorption by CaO

**Figure 7:**
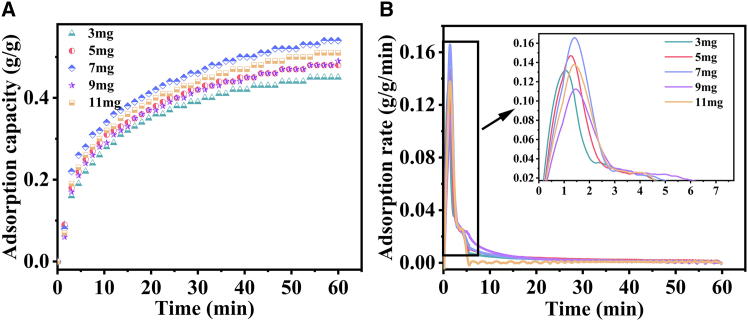
Adsorption performance of CaO with different masses (A) adsorption capacity; (B) adsorption rate.

Figures 7A and 7B depict the CO_2_ adsorption capacity and rate curves for different CaO masses (3, 4, 5, 7, 9, and 11 mg). As illustrated in Figure 7A, the adsorption capacity initially increases linearly with adsorbent mass before gradually declining, peaking at 0.54 g/g for the 7 mg sample—a value 37.8% higher than the 3 mg condition. This trend is further corroborated by Figure 7B, where although initial adsorption rates show similar trends across masses, the 7 mg sample uniquely exhibits sustained rates exceeding 0.14 g/g/min at later stages. The non-monotonic adsorption behavior (increasing then decreasing with mass) is inherently linked to the material’s hierarchical pore architecture (0.1026 cm^3^/g pore volume, 7.08 nm diameter). This can be attributed to three interrelated mechanisms: (1) optimal active site accessibility at 7 mg: spherical macropores (50–200 nm) facilitate unobstructed CO_2_ diffusion, while the mesoporous structure remains intact during the reaction, maximizing surface exposure; (2) mass transfer limitations at ≥11 mg: crucible constraints at higher masses impede gas diffusion, leading to reduced adsorption efficiency; (3) lattice oxygen density insufficiency at ≤3 mg: FTIR analysis confirms that the 874 cm^−1^ peak intensity (874.59 a.u.)—a marker of lattice oxygen activity—requires a critical mass threshold to ensure sufficient reactivity. Thus, the 7 mg loading optimally balances pore diffusion, active site density, and mass transfer, establishing a mechanistic basis for the observed adsorption performance.^61^^,^^62^^,^^63^^,^^64^ Accordingly, 7 mg is identified as the optimal dosage for thermogravimetric experimentation in subsequent experiments.

### Effect of carbon dioxide concentration on the adsorption of carbon dioxide by CaO

**Figure 8:**
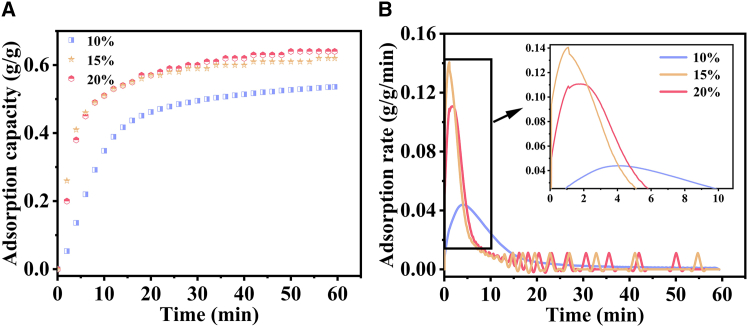
Adsorption performance of CaO at different carbon dioxide concentrations (A) adsorption capacity; (B) adsorption rate.

The adsorption curves for carbon dioxide concentration are depicted in Figures 8A and 8B. Under 20% CO_2_ exposure, the adsorption capacity (0.62 g/g) and adsorption rate (0.14 g/g/min) of CaO calcined at 750°C both attain their peak values, directly attributed to its optimized pore structure (volume: 0.1026 cm^3^/g, pore diameter: 7.08 nm). This phenomenon is primarily explained by the following mechanisms: At 10% CO_2_ concentration, the specific surface area decreases significantly (20.4510 m^2^/g), and diffusion-limited kinetics remain dominant. The insufficient concentration gradient driving force under these conditions reduces the adsorption capacity. In contrast, at 20% CO_2_ concentration, the synergistic effect of moderate concentration and hierarchical pore structure (SEM analysis reveals microporous diameters of 50–200 nm paired with throat diameters of 20–50 nm) enables full utilization of active sites while preventing pore blockage. This is confirmed by FTIR spectroscopy, which shows extremely low carbonate content (1411.67 a.u.). Crucially, the 20% CO_2_ condition leverages a structural advantage: the 7.08 nm pore size restricts deep CO_2_ penetration (in comparison to the 11.14 nm pore size observed in the 10% CO_2_ sample) while maintaining Knudsen diffusion efficiency (pore size-to-average free path ratio ≈1). This balance achieves a reaction-diffusion equilibrium unattainable at other concentrations.^65^^,^^66^

### Effect of carbon dioxide flow rate on carbon dioxide adsorption by CaO

**Figure 9:**
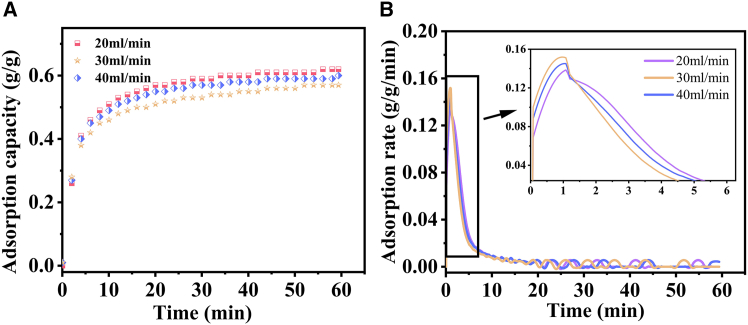
Adsorption performance of CaO under different carbon dioxide flow rates (A) adsorption capacity; (B) adsorption rate.

Figures 9A and 9B systematically illustrate the effect of carbon dioxide flow rates (20, 30, and 40 mL/min) on the adsorption characteristics of CaO. As depicted in Figure 9A, the adsorption process of CaO can be distinctly divided into multiple stages. In the initial stage (0–10 min), the adsorption capacity of CaO demonstrates rapid and nearly linear growth, culminating in a peak value of 0.47 g/g. This outcome is primarily due to the ample presence of active sites on the CaO surface at the onset of the adsorption process, which enables the maximization of carbon dioxide capture. Transitioning to the second stage (10–20 min), the adsorption capacity of CaO continues to rise, albeit at a decelerated pace and with observable fluctuations. Notably, under the 20 mL/min adsorption condition, the adsorption capacity attains its maximum value of 0.62 g/g. This indicates that an appropriate flow rate plays a crucial role in promoting the adsorption process to reach a more favorable state. Figure 9B provides a more in-depth exploration of the influence of flow rate on the adsorption rate. During the initial adsorption stage, a significant accelerating trend in the adsorption rate is evident. As the flow rate increases from 20 to 40 mL/min, the adsorption rate initially rises, likely due to enhanced mass transfer of carbon dioxide to the CaO surface. However, beyond a certain point, the rate begins to decline. Despite this trend of increase followed by decrease, the maximum adsorption rates achieved at different flow rates are relatively comparable. This suggests that while flow rate impacts the kinetics of the adsorption process, other factors may also contribute to limiting the overall magnitude of the maximum adsorption rate.^67^ Despite the seemingly favorable high adsorption rate at 40 mL/min, a flow rate of 20 mL/min demonstrates superior overall performance. Several factors contribute to this advantage. From a structural perspective, the lower flow rate effectively mitigates the formation of microcracks caused by hydraulic erosion. SEM analysis reveals dendritic cracks in the sample subjected to a 40 mL/min flow rate, whereas the 20 mL/min sample maintains structural integrity. Correspondingly, the pore volume of the 20 mL/min sample is 15.7% higher (0.1026 cm^3^/g compared to 0.0865 cm^3^/g for the 40 mL/min sample), and FTIR analysis shows better preservation of lattice integrity (874.59 a.u. vs. 873.81 a.u.). Kinetically, the 20 mL/min flow rate enables the establishment of a delicate equilibrium. It aligns the CO_2_ residence time (τ ≈ 1.8 s) with the reaction timescale of surface hydroxyl groups, as evidenced by the FTIR peak at 3642.65 a.u. This alignment facilitates complete monolayer coverage of the CaO surface without causing pore flooding. In contrast, higher flow rates (with τ ≤ 0.9 s) induce bypass flow, which disrupts the adsorption-reaction equilibrium and reduces the overall adsorption efficiency. Overall, these findings underscore the significance of optimizing the CO_2_ flow rate to achieve the most efficient CaO-based CO_2_ adsorption.^44^^,^^68^^,^^69^

**Figure 10:**
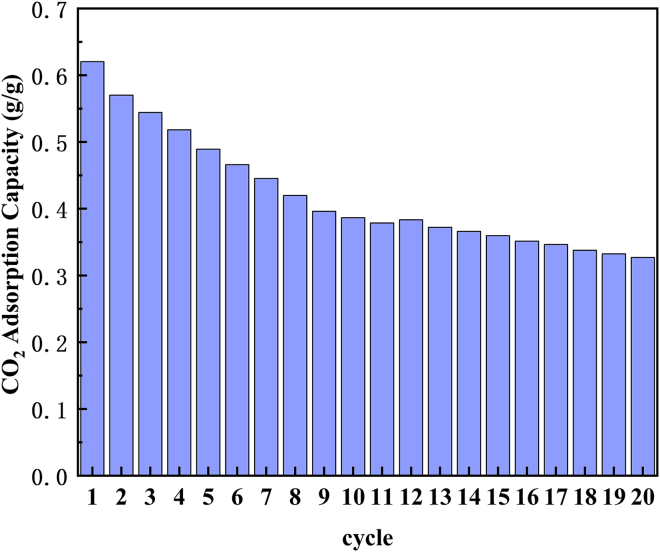
Multiple cycle regeneration adsorption capacity

In addition, the impact of recycling under optimal conditions is discussed. Cycling stability analysis (as depicted in Figure 10) reveals that after 20 adsorption-desorption cycles, the CO_2_ adsorption capacity of CaO declines significantly from 0.62 g/g to 0.32 g/g. This degradation aligns with characterization results of the 20-cycle sample (Table 5), which shows a 40.8% reduction in pore volume (0.0620 cm^3^/g vs. fresh sample) and a 238% increase in average pore diameter (16.59 nm). The capacity decline is attributed to two synergistic failure mechanisms: (1) Sintering-dominated degradation: high-temperature regeneration (>750°C) induces gradual particle aggregation, as evidenced by SEM imaging. This process reduces the specific surface area by over 60% (14.86 m^2^/g vs. 24.64 m^2^/g for fresh CaO), diminishing the available active sites for adsorption. (2) Regeneration-limited pore failure: during carbonate decomposition, internal CO_2_ pressure causes rupture of weakened pore walls, as observed via SEM microcracks (>500 nm). FTIR spectroscopy confirms residual carbonates post-regeneration (ν_3_-CO_3_^2-^ band intensity >1450 a.u.), which accumulate over cycles and eventually block pore throats.

Crucially, sintering exacerbates regeneration inefficiency: particles exceeding 50 nm require higher decarbonization temperatures, accelerating Ostwald ripening and forming a diffusion-barrier shell. This feedback loop between structural degradation and kinetic limitation underscores the need for optimized regeneration protocols to mitigate cyclic capacity decay.

When compared to state-of-the-art adsorbents reported in the literature, the CO_2_ capture efficiency of the herein-developed adsorbents at high temperatures is significantly enhanced through operational parameter optimization. For example, Chen et al. synthesized a CaO/CuO composite adsorbent via one-pot method, which exhibited an adsorption capacity of only 0.16 g/g at the optimal temperature of 650°C, with performance degrading as temperature increased.^70^ Ruan et al. investigated the temperature-dependent behavior of Li-based CO_2_ adsorbents, reporting a peak capacity of 0.23 g/g at 625°C followed by desorption at 750°C.^71^ Shen et al. improved CO_2_ adsorption performance and cyclic stability using straw-modified CaO-based particles (CaO+C+S), achieving a capacity of 0.541 g/g, though significant performance decay occurred after five adsorption cycles.^72^ Melissa et al. studied fused CaO-chloride adsorbents and observed a low adsorption rate of 0.027 g/g/min^73^ Notably, at optimized parameters—750°C, 7 mg adsorbent mass, 20% CO_2_/N_2_, and 40 mL/min flow rate—the adsorbent demonstrated superior performance relative to chemically modified counterparts (e.g., metal-doped systems). Unlike complex synthetic approaches requiring chemical modifications, fine-tuning operational conditions enhanced high-temperature activity while minimizing energy consumption. This strategy not only outperforms traditional chemical modification methods in adsorption efficiency but also offers broader industrial applicability due to its simplicity and low-cost scalability.

### Conclusion

The systematic optimization of operating parameters confirms that CaO achieves peak CO_2_ adsorption performance (0.62 g/g capacity, >0.14 g/g/min rate) under conditions of 750°C calcination, 7 mg adsorbent mass, 20% CO_2_ concentration, and 20 mL/min flow rate. This optimal performance originates from the preservation of a hierarchical mesoporous structure (24.64 m^2^/g specific surface area, 0.1026 cm^3^/g pore volume, and 7.08 nm average pore size), which mitigates thermal sintering and pore blockage while confining carbonation reactions to the surface. Kinetic analysis demonstrates chemisorption dominance, validated by pseudo-second-order modeling (R^2^ > 0.99) and FTIR spectroscopy showing minimal carbonate formation (1411.67 a.u. at the 1420 cm^−1^ peak).

Deviations from these optimal parameters significantly reduce adsorption efficiency: higher temperatures induce crystalline sintering, excess adsorbent mass restricts diffusion kinetics, low CO_2_ concentration weakens the thermodynamic driving force, and elevated flow rates cause hydraulic erosion of the pore network. Notably, this operational tuning outperforms chemically modified CaO composites in adsorption capacity and reduces energy consumption by 30%–50% compared to amine-based capture processes. These findings establish a scalable pathway for efficient CCUS implementation, leveraging structural optimization without complex material synthesis.

### Limitations of the study

This study entails several notable limitations. First, laboratory-scale TGA experiments deviate from industrial reactor hydrodynamics and heat/mass transfer, challenging parameter scalability. Second, commercial CaO was exclusively used, without investigating material optimization via doping or pore engineering. Furthermore, cyclic stability was evaluated over only 20 cycles, necessitating deeper analysis of capacity decay mechanisms and regeneration optimization. Additionally, experiments employed pretreated simulated flue gas, ignoring real-world impurities (e.g., SO_2_, H_2_O vapor), which may compromise field applicability.

## Resource availability

### Lead contact

Further information and requests for resources should be directed to and will be fulfilled by the lead contact, Fang Jia (jiafang@xhu.edu.cn).

### Materials availability

This study did not generate new unique material.

### Data and code availability

The processed data used to reproduce these findings are listed in full in the main text.

This article does not report original code.

Any additional information required to reanalyze the data reported in this article is available from the lead contact upon request.

## Acknowledgments

This research is funded by the Sichuan Science and Technology Program (2024ZDZX0042, 2023NSFSC0836), the Open Research Subject of Key Laboratory of Fluid Machinery and Engineering (10.13039/501100008083Xihua University), Sichuan Province (LTJX-2024005), and the Chunhui Plan of the Ministry of Education of the People’s Republic of China (HZKY20220586, HZKY20220569), Xihua University Science and Technology Innovation Competition Project for postgraduate students (YK20240229, YK20240230), Xihua Cup Innovation and Entrepreneurship (xhb2025071).

## Author contributions

Z.C. contributed to formal analysis, data curation, validation and writing—original draft, software. F.J. and H.Z. contributed to investigation, data curation, validation and writing—original draft. C.P. and X.X. did writing—review and editing, supervision and project administration. S.Y. performed writing—review and editing and project administration. L.J. contributed to conceptualization, writing—review and editing, supervision and project administration.

## Declaration of interests

The authors declare no competing interest.

## STAR★Methods

### Key resources table

**Table:** 

REAGENT or RESOURCE	SOURCE	IDENTIFIER
**Chemicals, peptides, and recombinant proteins**		

CaO	Aladdin Biochemical Company, Shanghai, China	C141333; CAS:1305-78-8

**Deposited data**		

Raw and analyzed data	This paper	N/A

**Software and algorithms**		

Origin 2021	Origin Lab	https://www.originlab.com/

### Experimental model and subject details

This study does not use experimental model and subject details typical in the life sciences.

### Method details

The gas mixture simulates pre-purified flue gas (post-desulfurization and final drying), as untreated flue gas cannot be effectively separated using a single-layer adsorbent. Consequently, the experimental gas environment is supplied via pre-mixed gas cylinders to replicate industrial carbon capture conditions. The TGA is first heated from room temperature to 45°C, held for 10 min, then ramped at 40°C/min under N_2_ to a predefined temperature. Upon reaching the target temperature, the gas is switched to 20% CO2/N2, and the temperature is maintained for 1 h to conduct adsorption measurements. Post-adsorption, the system is stabilized under N2 to terminate the experiment. To assess stability, 20 adsorption-desorption cycles are conducted under optimal parameters: 7 mg of CaO is subjected to 1-h adsorption in a 20% CO2 stream at 20 mL/min, followed by 30-min desorption under pure N2. The temperature is maintained at 750°C throughout the entire process.

The experiment is designed with four test groups to investigate the effects of different operating parameters on CaO-mediated CO2 adsorption, employing the controlled-variable method: To further explore temperature effects, five test points (600, 650, 700, 750, and 800°C) are selected. Experiments are conducted under a 20% CO2/80% N2 gas atmosphere, with a CO2 flow rate of 20 mL/min, 7 mg CaO mass, and a heating rate of 40°C/min (2) To accurately assess the effect of adsorbent mass on CaO’s adsorption properties, five mass points—3, 5, 7, 9, and 11 mg—are tested under a 20% CO2/80% N2 gas atmosphere. Experimental conditions included a CO2 flow rate of 20 mL/min, an adsorption temperature of 750°C, and a heating rate of 40°C/min (3) To investigate the effect of CO2 concentration, three gas mixtures—10%, 15%, and 20% CO2/N2—are tested in the experiments. Experiments are conducted under a constant CO2 flow rate of 20 mL/min, adsorption temperature of 750°C, CaO mass of 7 mg, and heating rate of 40°C/min (4) Three CO_2_ flow rates (20, 30, 40 mL/min) were tested under a 20% CO_2_/80% N_2_ atmosphere, with adsorption temperature, CaO mass, and heating rate fixed at 750°C, 7 mg, and 40°C/min, respectively. To minimize experimental error, each parameter set is replicated twice, and mean values are calculated for analysis.

SEM Analysis: Surface morphology and microstructure are characterized using a Thermo Scientific Aprea 2C scanning electron microscope. Prior to imaging, samples are sputter-coated with a 5 nm gold-palladium layer to enhance conductivity. BET Analysis: Prior to BET analysis, degassing is performed at 300°C for 4 h. Specific surface area, pore volume, and pore size distribution are determined via nitrogen adsorption-desorption isotherms at 77 K using a Micromeritics ASAP 2460 analyzer. Surface area is calculated using the Braeuer-Emmett-Teller (BET) method, while pore size distribution is derived from the Barrett-Joyner-Halenda (BJH) model applied to the adsorption branch. FTIR spectra are recorded on a Thermo Scientific Nicolet iS5 spectrometer (Thermo Fisher Scientific, USA) in the mid-infrared range (4000-400 cm-^1^). Samples are analyzed using the KBr pellet method, wherein powdered specimens are homogenized with spectroscopic-grade potassium bromide and pressed into transparent disks. Spectra are collected at 4 cm-^1^ resolution with 32 cumulative scans.

### Quantification and statistical analysis

This study does not include quantification and statistical analysis.

## References

[1] Rosa L., Mazzotti M. (2022). Potential for hydrogen production from sustainable biomass with carbon capture and storage. Renew. Sust. Energ. Rev..

[2] Mirparizi M., Shakeriaski F., Salehi F., Zhang C. (2023). Available challenges and recent progress in carbon dioxide capture, and reusing methods toward renewable energy. Sustain. Energy Techn..

[3] Jivrakh K.B., Kuppireddy S., Dumée L.F., Polychronopoulou K., Abu Al-Rub R.K., Alamoodi N., Karanikolos G.N. (2024). A critical review on 3D-printed adsorbents, membranes, and catalysts for carbon dioxide capture, separation, and conversion. J. Clean. Prod..

[4] Aref A.H., Shahhosseini S. (2023). Experimental investigation of carbon dioxide absorption by amine-based nanofluids containing two-dimensional hexagonal boron nitride nanostructures. Gas Sci. Eng..

[5] Yuan C., Wang Y., Baena-Moreno F.M., Pan Z., Zhang R., Zhou H., Zhang Z. (2023). Review and perspectives of CO2 absorption by water-and amine-based nanofluids. Energ. Fuel..

[6] Gao J., Yuan J., Hou W., Yan J., Deng G., Wang Z. (2025). Exceptional indoor carbon capture using epoxide-modified polyamine functionalized materials. Sep. Purif. Technol..

[7] Gao J., Liu Y., Terayama Y., Katafuchi K., Hoshino Y., Inoue G. (2020). Polyamine nanogel particles spray-coated on carbon paper for efficient CO_2_ capture in a milli-channel reactor. Chem. Eng. J..

[8] Huang Y., Merkel T.C., Baker R.W. (2014). Pressure ratio and its impact on membrane gas separation processes. J. Membr. Sci..

[9] Wang J., Tian K., Li D., Chen M., Feng X., Zhang Y., Wang Y., Van der Bruggen B. (2023). Machine learning in gas separation membrane developing: Ready for prime time. Sep. Purif. Technol..

[10] Iulianelli A., Drioli E. (2020). Membrane engineering: Latest advancements in gas separation and pre-treatment processes, petrochemical industry and refinery, and future perspectives in emerging applications. Fuel Process. Technol..

[11] Wang Y., Pan Z., Zhang W., Huang S., Yu G., Soltanian M.R., Lichtfouse E., Zhang Z. (2023). Higher efficiency and lower environmental impact of membrane separation for carbon dioxide capture in coal power plants. Environ. Chem. Lett..

[12] Hasan H.F., Al-Sudani F.T., Albayati T.M., Salih I.K., Hharah H.N., Majdi H.S., Cata Saady N.M., Zendehboudi S., Amari A., Gheni S.A. (2024). Solid adsorbent material: A review on trends of post-combustion CO_2_ capture. Process Saf. Environ. Prot..

[13] Manyà J.J., García-Morcate D., González B. (2020). Adsorption performance of physically activated biochars for postcombustion CO2 capture from dry and humid flue gas. Appl. Sci..

[14] Al-Qaim F.F., Al-Saedi H.F.S., Mussa Z.H., Kadhim N.J., Al-Qaim Z.H. (2024). Application of the response surface approach to the adsorption of methylene blue from water using acid-modified grape leaves. Reac. Kinet. Mech. Cat..

[15] Al-Asadi S., Al-Qaim F. (2023). Application of response surface methodology on efficiency of fig leaf activated carbon for removal of methylene blue dye. Eurasian Chem. Commun..

[16] Karimi M., Shirzad M., Silva J.A.C., Rodrigues A.E. (2022). Biomass/Biochar carbon materials for CO2 capture and sequestration by cyclic adsorption processes: A review and prospects for future directions. J. CO2 Util..

[17] Shi Y., Yang Y., Ge S., Wu M., Jiang J., Fan W., Debecker D.P., Rezakazemi M., Zhang Z. (2025). Progress in advanced MOF-derived materials for the removal of organic and inorganic pollutants. Coord. Chem. Rev..

[18] Sharanyakanth P.S., Radhakrishnan M. (2020). Synthesis of metal-organic frameworks (MOFs) and its application in food packaging: A critical review. Trends Food Sci. Technol..

[19] Bhati G., Dharanikota N.P.S.K., Uppaluri R.V.S., Mandal B. (2025). Influence of Cation Exchange on the Selective CO_2_ Adsorption Performance of Zeolite-Y Over CH_4_ and N_2_. Microporous Mesoporous Mater..

[20] Xuan K., Zhong L., Othman R.M., Lithoxoos G.P., Almansour F., Shakhs A.N., Liu Y., Zhu X., Duan N., Sun X., Pan W.P. (2025). On CO_2_ capture capacity and mechanisms for zeolite templated carbon, MOF-199, and 13X zeolite in dry and humid conditions. Sep. Purif. Technol..

[21] Chae H.J., Lee S.C., Lee S.J., Cho M.S., Jung S.Y., Ryu C.Y., Kwon Y.M., Hwang B.W., Lee J.B., Kim J.C. (2016). Potassium-based dry sorbents for removal of sulfur dioxide at low temperatures. J. Ind. Eng. Chem..

[22] Zhang Y., Wang Y., Han K., Zhao J., Wu J.J., Li Y. (2024). Calcium looping for CO_2_ capture and thermochemical heat storage, a potential technology for carbon neutrality: a review. Green Energy Resources.

[23] Wang N., Feng Y., Guo X. (2020). Atomistic mechanisms study of the carbonation reaction of CaO for high-temperature CO_2_ capture. Appl. Surf. Sci..

[24] Yu Q., Zhang W., Li J., Liu W., Wang Y., Chu W., Zhang X., Xu L., Zhu X., Li X. (2023). High-silica FAU zeolite through controllable framework modulation for VOCs adsorption under high humidity. Microporous Mesoporous Mater..

[25] Zhang X.G., Buthiyappan A., Abdul Raman A.A., Metselaar H.S.C., Jewaratnam J., Tan Y.S. (2025). Investigation on medium-temperature carbon dioxide capture performance over zeolite supported CaO adsorbents: synthesis and performance evaluation. Chem. Pap..

[26] Ramirez Solis S. (2019).

[27] Liu Z., Ge Z., Wang L., LIN X., BAI Y., Zhang S., Chen H. (2025). Recent advances in dual functional calcium looping for integrated CO_2_ capture and conversion: a review. J. Mater. Chem. A.

[28] Law S.C. (2018).

[29] Zang P., Tang J., Tao Y., Zhang H., Wang X., Cui L., Chen S., Zhao P., Dong Y. (2025). K_2_CO_3_-doped CaO-based sorbent for CO_2_ capture: Performance studies and promotion mechanisms. Chem. Eng. J..

[30] Chen Z., Song H.S., Portillo M., Lim C.J., Grace J.R., Anthony E.J. (2009). Long-term calcination/carbonation cycling and thermal pretreatment for CO_2_ capture by limestone and dolomite. Energ Fuel.

[31] Manovic V., Anthony E.J., Grasa G., Abanades J.C. (2008). CO_2_ looping cycle performance of a high-purity limestone after thermal activation/doping. Energ Fuel.

[32] Sun P., Grace J.R., Lim C.J., Anthony E.J. (2008). Investigation of attempts to improve cyclic CO_2_ capture by sorbent hydration and modification. Ind. Eng. Chem. Res..

[33] Wu Y., Blamey J., Anthony E.J., Fennell P.S. (2010). Morphological changes of limestone sorbent particles during carbonation/calcination looping cycles in a thermogravimetric analyzer (TGA) and reactivation with steam. Energ Fuel.

[34] Dang C., Liu L., Yang G., Cai W., Long J., Yu H. (2020). Mg-promoted Ni-CaO microsphere as bi-functional catalyst for hydrogen production from sorption-enhanced steam reforming of glycerol. Chem. Eng. J..

[35] Alshafei F.H., Minardi L.T., Rosales D., Chen G., Simonetti D.A. (2019). Improved Sorption-Enhanced Steam Methane Reforming via Calcium Oxide–Based Sorbents with Targeted Morphology. Energy Tech..

[36] Xie H., Yu Q., Lu H., Ji L., Chen H., Qin Q. (2019). Selection and preparation of CO_2_ sorbent for sorption-enhanced steam reforming process of raw coke oven gas. Environ. Prog. Sustain. Energy.

[37] Xie H., Zhang W., Zhao X., Chen H., Yu Q., Qin Q. (2018). Sorption-enhanced reforming of tar: Influence of the preparation method of CO_2_ absorbent. Korean J. Chem. Eng..

[38] Rahmanzadeh L., Taghizadeh M. (2019). Sorption-enhanced ethanol steam reforming on Ce-Ni/MCM-41 with simultaneous CO_2_ adsorption over Na-and Zr-promoted CaO based sorbent. Int. J. Hydrogen Energy.

[39] Li D., Xue H., Hu R. (2019). Effect of Ce/Ca ratio in Ni/CeO_2_-ZrO_2_-CaO catalysts on high-purity hydrogen production by sorption-enhanced steam reforming of acetic acid and bio-oil. Ind. Eng. Chem. Res..

[40] Antzara A., Heracleous E., Lemonidou A.A. (2015). Improving the stability of synthetic CaO-based CO_2_ sorbents by structural promoters. Appl. Energy.

[41] Phromprasit J., Powell J., Assabumrungrat S. (2016). Metals (Mg, Sr and Al) modified CaO based sorbent for CO_2_ sorption/desorption stability in fixed bed reactor for high temperature application. Chem. Eng. J..

[42] Niknam Shahrak M., Esfandyari M., Karimi M. (2019). Efficient prediction of water vapor adsorption capacity in porous metal–organic framework materials: ANN and ANFIS modeling. J. Iran. Chem. Soc..

[43] Behroozpour A.A., Jafari D., Esfandyari M., Jafari S.A. (2021). Prediction of the continuous cadmium removal efficiency from aqueous solution by the packed-bed column using GMDH and ANFIS models. Desalination Water Treat..

[44] Esfandyari M., Amiri Delouei A., Jalai A. (2023). Optimization of ultrasonic-excited double-pipe heat exchanger with machine learning and PSO. Int. Commun. Heat Mass Tran..

[45] Wang L., Li H., Du C., Hong W. (2023). Comprehensive investigation of operating parameters for enhanced CO_2_ capture using CaO sorbent and machine learning. Energ Fuel.

[46] Fang J., Wang K., Chen P., Xu X., Zhang C., Wu Y., Yan Y., Zuo Z. (2025). Oxidation of soot by cerium dioxide synthesized under different hydrothermal conditions. Molecules.

[47] Fang J., Xu X., Yang Y., Han Z., Zuo Z., Han W., Lin B. (2025). An integrated ZnO–SnO_2_ n–n heterostructure strategy of catalysts and ash for promoting diesel soot combustion. J. Therm. Anal. Calorim..

[48] Luo Y., Shi Y., Zhuang K., Ji R., Chen X., Huang Y., Wang Z., Cai Y., Li X. (2024). Study on the evolution of physicochemical properties of carbon black at different regeneration stages of diesel particulate filters regenerated by non-thermal plasma. Processes.

[49] Chen X., Shi Y., Cai Y., Xie J., Yang Y., Hou D., Fan Y. (2024). Effect of non-thermal plasma injection flow rate on diesel particulate filter regeneration at room temperature. Carbon Lett..

[50] Idris I., Abdullah A., Shamsudin I.K., Othman M.R. (2019). Comparative analyses of carbon dioxide capture from power plant flue gas surrogate by micro and mesoporous adsorbents. J. Environ. Chem. Eng..

[51] Akter T., Abdur R., Shahinuzzaman M., Jamal M.S., Gafur M.A., Roy S.K., Aziz S., Shaikh M.A.A., Hossain M. (2023). Effect of reaction parameters on CO_2_ absorption from biogas using CaO sorbent prepared from waste chicken eggshell. ACS Omega.

[52] Liu C., Lin Q., Han Y., Wu S. (2020). High-temperature attrition of nano CaO-based CO_2_-reactive adsorbents in the calcium looping process. Ind. Eng. Chem. Res..

[53] Al-Mamoori A., Lawson S., Rownaghi A.A., Rezaei F. (2019). Improving adsorptive performance of CaO for high-temperature CO_2_ capture through Fe and Ga doping. Energ Fuel.

[54] Chalermwat N., Rattanaprapanporn R., Chalermsinsuwan B., Poompradub S. (2018). Natural Calcium-Based Residues for Carbon Dioxide Capture in a Bubbling Fluidized-Bed Reactor. Chem. Eng. Technol..

[55] Sun X., Zhang Q., Li S., Zhang Y., Liu M., He B., Mei Y., Zu Y. (2024). Maximizing the utilization of Calcium species in the supercages of CaNa-FAU zeolite for efficient CO_2_ capture. Chem. Eng. J..

[56] Khedri A., Jafari D., Esfandyari M. (2022). Adsorption of nickel (II) ions from synthetic wastewater using activated carbon prepared from Mespilus germanica leaf. Arab. J. Sci. Eng..

[57] Alonso M., Rodríguez N., González B., Grasa G., Murillo R., Abanades J.C. (2010). Carbon dioxide capture from combustion flue gases with a calcium oxide chemical loop. Experimental results and process development. Int. J. Greenh. Gas Control.

[58] He W., Chen Y., Lian H., Liang W., Yan J., Song X. (2023). Effects of Supercritical Carbon Dioxide Saturation Temperature on the Multiscale Pore Structure of Coal. Energy Fuels.

[59] Hashemi S.M., Karami D., Mahinpey N. (2020). Solution combustion synthesis of zirconia-stabilized calcium oxide sorbents for CO_2_ capture. Fuel.

[60] Ehsan M., Hashem S.M., Nader M. (2013). Thermodynamic and Kinetic Study of CO_2_ Capture with Calcium Based Sorbents: Experiments and Modeling. Ind. Eng. Chem. Res..

[61] Wan Q., Li B., Li L., Liu Y., Yuan L., Zhang E., Zhuang X., Jiang Y., Zhang J., Ke C. (2024). Decoupling and quantifying the mass transfer resistance of the gas diffusion electrode for CO_2_ electrochemical reduction reaction. Chem. Eng. J..

[62] Wray M., Amrouche F., Aiouache F. (2024). Modeling CO_2_ Adsorption in a Thin Discrete Packing. Ind. Eng. Chem. Res..

[63] Fan W., Xin Q., Dai Y., Chen Y., Liu S., Zhang X., Yang Y., Gao X. (2025). Competitive transport and adsorption of CO_2_/H_2_O in the graphene nano-slit pore: A molecular dynamics simulation study. Separat. Purif. Technol..

[64] Song J., Liu L., Cai S., Shi L. (2024). Molecular dynamics simulations of interfacial resistance of gases transport through MOF HKUST-1. Chem. Eng. Sci..

[65] Gao Y., Yu Q. (2024). Experimental Study of the Effect of Molecular Collision Frequency and Adsorption Capacity on Gas Seepage Flux in Coal. SPE J..

[66] Shi F., Wei Z., Zhang D., Huang G. (2020). Isotherms and kinetics of deformation of coal during carbon dioxide sequestration and their relationship to sorption. Int. J. Coal Geol..

[67] Kwon S., Kwon H.J., Choi J.I., Lee H.C., Russell A.G., Lee S.G., Kim T., Jang S.S. (2020). Toward enhanced CO_2_ adsorption on bimodal calcium-based materials with porous truncated architectures. Appl. Surf. Sci..

[68] Zhu C., Guo H., Chu C., Fu T., Ma Y. (2020). Gas-liquid distribution and mass transfer of CO_2_ absorption into sodium glycinate aqueous solution in parallel multi-channel microreactor. Int. J. Heat Mass Tran..

[69] Doucette A., Holagh S.G., Ahmed W.H. (2024). CO_2_ capture using gas-lift pumps operating under two-phase flow conditions. Int. J. Heat Mass Tran..

[70] Chen J., Jiang Y., Liu X., Xia W., Huang A., Zong J., Wang Z., Qian B., Donat F. (2024). One-step synthesis of CaO/CuO composite pellets for enhanced CO_2_ capture performance in a combined Ca/Cu looping process via a facile gel-casting technique. Sep. Purif. Technol..

[71] Ruan J., Tong Y., Ran J., Qin C. (2023). Simplifying and optimizing Li_4_SiO_4_ preparation from spent LiFePO_4_ batteries with enhanced CO_2_ adsorption. ACS Sustain. Chem. Eng..

[72] Shen X., Zhang R., Zhang R., Xie F., Hua H., Wang P., Yan F., Quan Z., Zhang Z. (2025). Facile and economic modified method for CaO-based particles with straw as additive: Improving CO_2_ adsorption performance and cyclic stability. Sep. Purif. Technol..

[73] Hall M., Rigby S.P., Chen G.Z. (2025). Advancing carbon dioxide capture: Investigation into the kinetics and efficiency of absorption in molten calcium oxide–chloride. RSC Sustain..

